# Exploratory application of machine learning methods on patient reported data in the development of supervised models for predicting outcomes

**DOI:** 10.1186/s12911-022-01973-9

**Published:** 2022-09-01

**Authors:** Deepika Verma, Duncan Jansen, Kerstin Bach, Mannes Poel, Paul Jarle Mork, Wendy Oude Nijeweme d’Hollosy

**Affiliations:** 1 Department of Computer Science, Norwegian University of Science and Technology, Trondheim, Norway; 2 Faculty of Electrical Engineering, Mathematics and Computer Science, University of Twente, Twente, The Netherlands; 3 Department of Public Health and Nursing, Norwegian University of Science and Technology, Trondheim, Norway; 4 eHealth Cluster, Roessingh Research and Development, Enschede, The Netherlands

**Keywords:** Machine learning, Low-back pain, Neck pain, Patient-reported outcomes, Self-reported measures, Outcome Prediction

## Abstract

**Background:**

Patient-reported outcome measurements (PROMs) are commonly used in clinical practice to support clinical decision making. However, few studies have investigated machine learning methods for predicting PROMs outcomes and thereby support clinical decision making.

**Objective:**

This study investigates to what extent different machine learning methods, applied to two different PROMs datasets, can predict outcomes among patients with non-specific neck and/or low back pain.

**Methods:**

Using two datasets consisting of PROMs from (1) care-seeking low back pain patients in primary care who participated in a randomized controlled trial, and (2) patients with neck and/or low back pain referred to multidisciplinary biopsychosocial rehabilitation, we present data science methods for data prepossessing and evaluate selected regression and classification methods for predicting patient outcomes.

**Results:**

The results show that there is a potential for machine learning to predict and classify PROMs. The prediction models based on baseline measurements perform well, and the number of predictors can be reduced, which is an advantage for implementation in decision support scenarios. The classification task shows that the dataset does not contain all necessary predictors for the care type classification. Overall, the work presents generalizable machine learning pipelines that can be adapted to other PROMs datasets.

**Conclusion:**

This study demonstrates the potential of PROMs in predicting short-term patient outcomes. Our results indicate that machine learning methods can be used to exploit the predictive value of PROMs and thereby support clinical decision making, given that the PROMs hold enough predictive power

**Supplementary Information:**

The online version contains supplementary material available at 10.1186/s12911-022-01973-9.

## Introduction

While the application of machine learning (ML) methods is expanding into new clinical areas, both in medical research and clinical practice [1, 2], these methods have rarely been used on patient-reported outcome measurements (PROMs). PROMs are used commonly for health conditions that are difficult to assess with objective measurements, such as non-specific musculoskeletal pain and mental disorders. The predictive capabilities of ML methods, combined with clinical expertise, may increase the precision of clinical decision-making and thereby improve patient outcomes in these conditions [3]. To the best of our knowledge, no prognostic models based on ML methods are currently in clinical use for predicting outcomes among patients with non-specific musculoskeletal conditions, such as neck pain and low back pain. These conditions are among the leading causes of disability worldwide [4] and improving the precision of clinical decision-making to improve patient outcomes will likely have a substantial impact on their disability burden.

Predicting outcomes from PROMs in patients with neck and/or low back pain (NLBP) is a challenging task owing to the subjective nature of the data. Nevertheless, some recent studies have shown promising results in applying ML methods. In a study by d’Hollosy et al. [5], binary classification models trained on PROMs data were used to predict whether low back pain patients should be referred to a Multidisciplinary biopsychosocial rehabilitation (MBR) program or undergo surgery. The authors concluded that the ML models show small to medium learning effects. Another study showed that a ML least shrinkage selection operator approach performs well in predicting pain-related disability at 2-year follow-up among older adults with NLBP [6].

The current study continues this line of research, intending to investigate to what extent different ML methods applied to PROMs data can identify predictors of outcomes and predict outcomes among patients with non-specific NLBP. The research question addressed in this work is: *Can Machine Learning methods make predictions using patient-reported data to facilitate the shared decision-making process for patients with NLBP?*

## Background

Early and thorough assessment of non-specific low back pain is recommended to support a clinician’s treatment planning for patients at increased risk of poor outcome [7]. MBR is a commonly used treatment approach that targets biological, psychological, and social influences on low back pain [8]. However, this treatment approach is costly and time-consuming and the decision on whether a patient should start an MBR program is challenging. Supported self-management via web or mobile application is another alternative treatment approach that has gained popularity in recent years [9]. One such decision support system (DSS) delivered via mobile application has been implemented in the selfBACK project [10]. selfBACK DSS was developed to facilitate, improve, and reinforce self-management of non-specific LBP. The core idea is to empower patients to take control of their symptoms and treatment.

PROMs are a valuable source of information but few studies have exploited PROMs in the context of applying ML methods. Rahman et al. [11] performed a study, aimed at predicting pain volatility among users of a supported self-management delivered via a mobile application (“Manage My Pain”). Unsupervised ML methods were used to cluster the users followed by supervised ML methods to predict pain volatility levels at 6-month follow-up using in-app PROMs (130 in total). The best accuracy was 70%, achieved using Random Forest. In a follow-up study, Rahman et al. [12] addressed the topic of identifying the most important predictors of pain volatility using different feature selection methods and found that similar prediction accuracy (68%) can be achieved using only a few predictors (9 features). In another study, Harris et al. [13] compared the performance of four supervised ML models including Logistic, LASSO, Gradient Boosting Machines, and Quadratic Discriminant Analysis for predicting whether or not a patient achieves a minimal clinically important difference (MCID) in several pain and function related outcomes at 1-year post knee arthroplasty. Using preoperative PROMs as predictors, they found that similar performance can be achieved across different models for various outcomes by varying the number of inputs. None of the models was found to be superior for all the outcomes. In contrast, Fontana et al. [6] found that LASSO performs better than Gradient Boosting Machines and Support Vector Machines in predicting MCID at 2-year follow-up among patients undergoing knee or hip arthroplasty. Similarly, Huber et al [14] compared the performance of eight supervised ML models for predicting MCID at six months among patients undergoing knee or hip replacement. Preoperative PROMs were used as predictors, and the results showed that Gradient Boosting machines yielded the most accurate prediction.

## Datasets

In this section we describe the two datasets used in this work to build classification and regression models for PROMs.

### Dataset 1

Dataset 1 consists of PROMs collected from LBP patients recruited in the intervention group of the selfBACK randomised controlled trial (RCT),^1^ which aimed at facilitating self-management among patient with non-specific LBP.

**Fig. 1:**
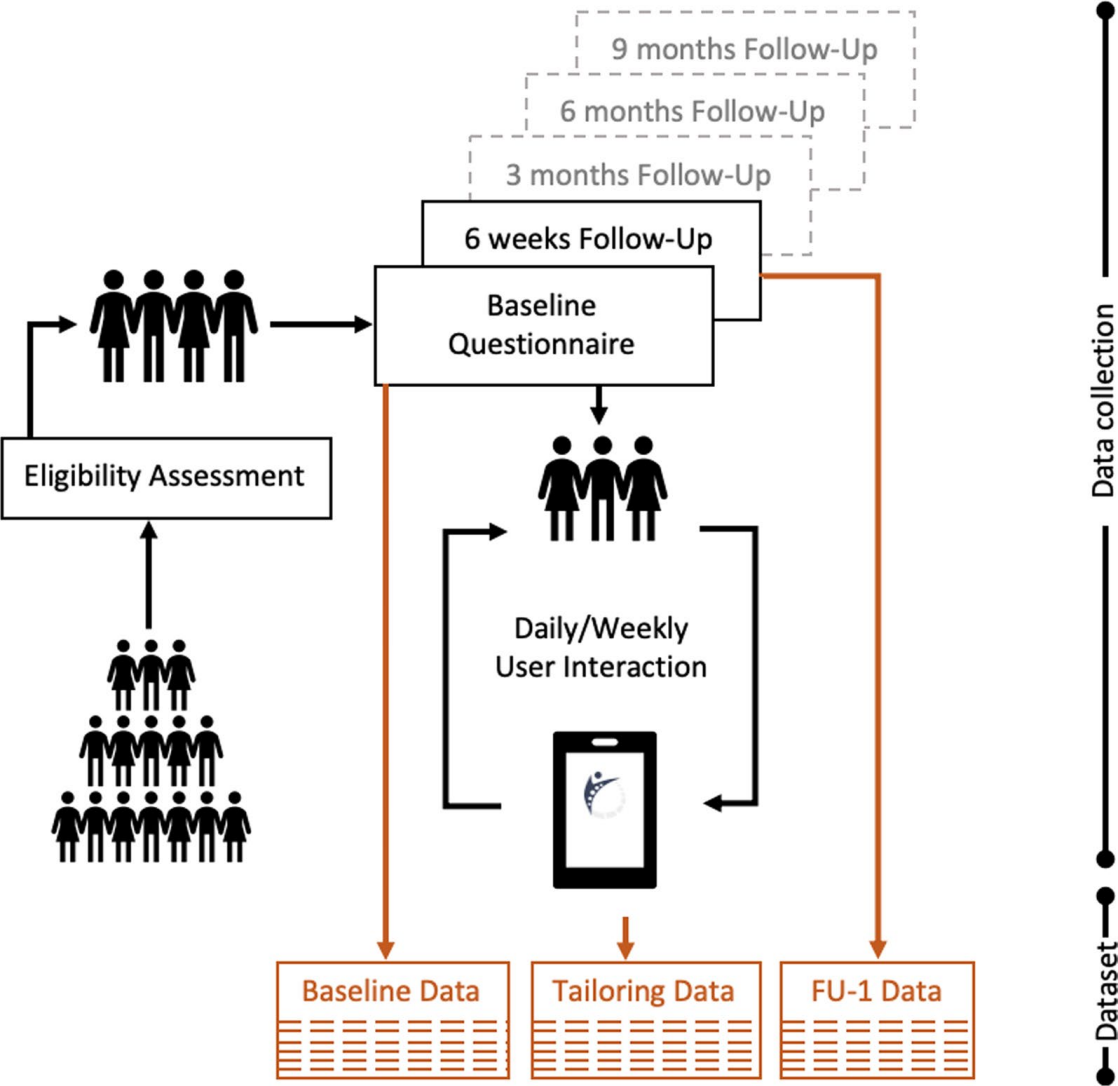
Overview of data collection in the selfBACK randomized controlled trial. The different data components are indicated by the orange boxes

Figure 1 shows the data collection in selfBACK. The data is categorised into Baseline, Tailoring and Follow-Up (FU) data. Patients were recruited through the referral of their primary care clinician, followed by screening for eligibility based on a set criteria. Eligible patients who accepted to join the study answered questionnaires at different time points: (1) at the time of intake: Baseline questionnaire (*Baseline Data*), (2) at the end of every week: Tailoring questionnaire (*Tailoring Data*), (3) at the end of 6-weeks, 3-months, 6-months, 9-months: Follow-Up questionnaire ((*FU Data*)). The questionnaire measures are:

- Pain level
- Pain self-efficacy
- Physical activity
- Sleep quality
- Fear avoidance
- Functional ability
- Work-ability
- Mood

The baseline questionnaire also included demographics (education, employment and family). The tailoring and follow-up questions are subsets of the baseline questions. A comprehensive overview of the data collection can be found in Sandal et al. [15].

Based on the patients’ responses at baseline, the selfBACK mobile application recommends an exercise plan and educational elements along with tracking their number of steps everyday from a wearable device (Xiaomi Mi Band 3). Exercise completion and educational readings were self-reported in the app. From this dataset, we only use the Baseline and FU-1 data for the experiments.

#### Target outcomes

The average pain (last week) and work-ability reported by the patients in the FU-1 dataset were chosen as target outcomes from *dataset 1*, referred to as $PA_{f}$ and $WAI_{f}$ respectively. Average pain is self-assessed using the Numerical Pain Rating Scale [16], ranging from 0(*no pain*) to 10(*(disabling) severe pain*). Pain rating scales are commonplace in the medical and healthcare context and are used widely in different medical environments as a tool of communicating or expressing level of pain experienced by an individual. Work Ability Index (WAI) [17] is a self-assessment measure used in workplace and occupational health surveys and uses the Numerical Rating Scale ranging from 0(*completely unable to work*) to 10(*workability at its best*). It is widely used in occupational health and research to facilitate understanding different dimensions of a working individual including their current ability to work compared with their lifetime best, self-prognosis of their work-ability in the last two years, their ability to work with respect to the demands of the job, the number of sick leaves taken in the last year, among others.

The dataset for predicting $PA_{f}$ contains completed data from 218 patients, while for predicting $WAI_{f}$ contains data from 159 patients. The number of patients is less in $WAI_{f}$ due to the exclusion of patients who did not answer the baseline WAI, among them are the retired patients as this measure does not apply to them. The final dataset comprises of 47 self-reported measures, which form the predictor variables.

### Dataset 2

**Fig. 2:**
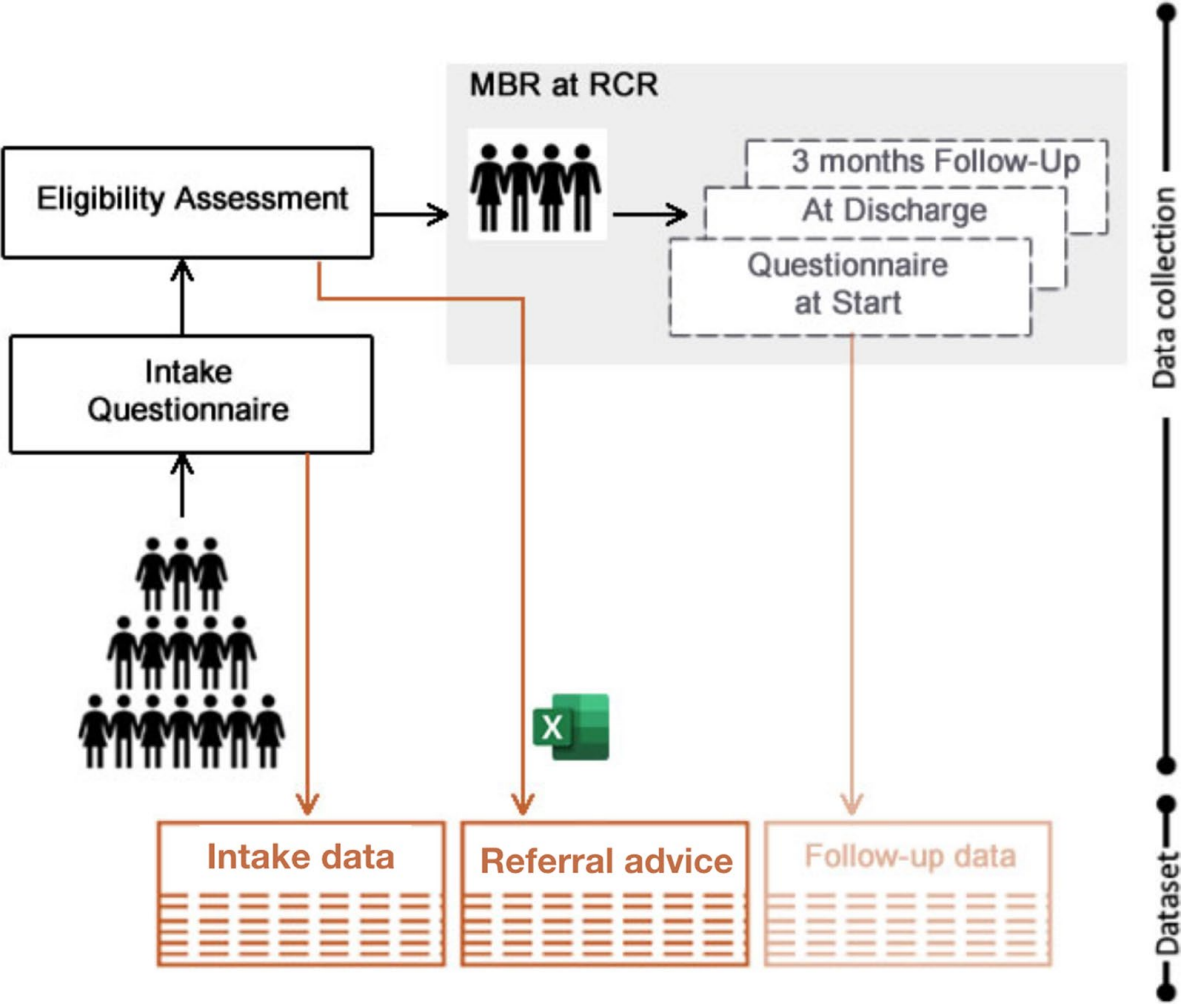
Overview of the assessment moments with questionnaires at the pain rehabilitation centre RCR, Enschede, the Netherlands. MBR: Multidisciplinary Biopsychosocial Rehabilitation

Data was collected by the Roessingh Center of Rehabilitation (RCR), Netherlands, between 2012–2019. The data consists of PROMs collected from NLBP patients referred to MBR using questionnaires administered at four time points: (1) before intake, (2) at the start, (3) at the end, and (4) after 3 months of pain rehabilitation, see Fig. 2. Patients gave consent to use their data for scientific research.

The questionnaires contain self-reported measures commonly used in pain rehabilitation,

- Hospital Anxiety and Depression Scale (HADS) [18]
- Multidimensional Pain Inventory (MPI) [19]
- Pain Disability Index (PDI) [20]
- Psychological Inflexibility in Pain Scale (PIPS) [21]
- Rand-36 Health Survey (RAND-36) [22]

The responses on the 121 questions were used to calculate 23 scores, shown in table 1. These scores are used as features in the ML experiment.

**Table 1:** The PROMs included in Dataset 2

*HADS*		
Anxiety	Depression	Total score
*MPI*		
Pain severity	Interference	Life control
Affective distress	Solicitous responses	Distracting responses
Punishing responses	Support	Household chores
Outdoor work	Social activities	General activities
*PDI*		
Total score		
*PIPS*		
Avoidance	Cognitive fusion	Total score
*RAND-36*		
Physical functioning	Role limitations	Vitality
Mental health		

#### Target outcome

The targets were the referral advice, which were given after the eligibility assessment (Fig. 2). The data set contained 1040 patient records. These records were labelled according to 4 possible referral advises:

- Clinic RCR (n = 235): accepted for MBR at the RCR and advised to follow a clinical treatment path.
- Polyclinic RCR (n = 294): accepted for MBR at the RCR and advised to follow a polyclinical treatment path.
- Polyclinic RMCR (n = 140): referred to Roessingh Medinello Center of Rehabilitation (RMCR), which is similar to Polyclinic RCR but provides treatment paths for less complicated patients.
- Reject (n = 371): referred to the RCR from primary or secondary care, but rejected after intake by clinician at RCR because they were not eligible.

This labelling resulted into an unbalanced dataset. The final dataset is shown in Table 2. The column ’# of cases’ shows the total number of cases (Class A + Class B) in Dataset 2 per combination.

**Table 2:** Referral combinations the classification algorithms were trained on

Model	Class A	Class B	# of cases
1	Clinic RCR	Polyclinic RCR	529
2	Clinic RCR	Reject	606
3	Polyclinic RCR	Reject	665
4	Polyclinic RMCR	Clinic RCR	375
5	Polyclinic RMCR	Polyclinic RCR	434
6	Polyclinic RMCR	Reject	511

## Methods

**Fig. 3:**
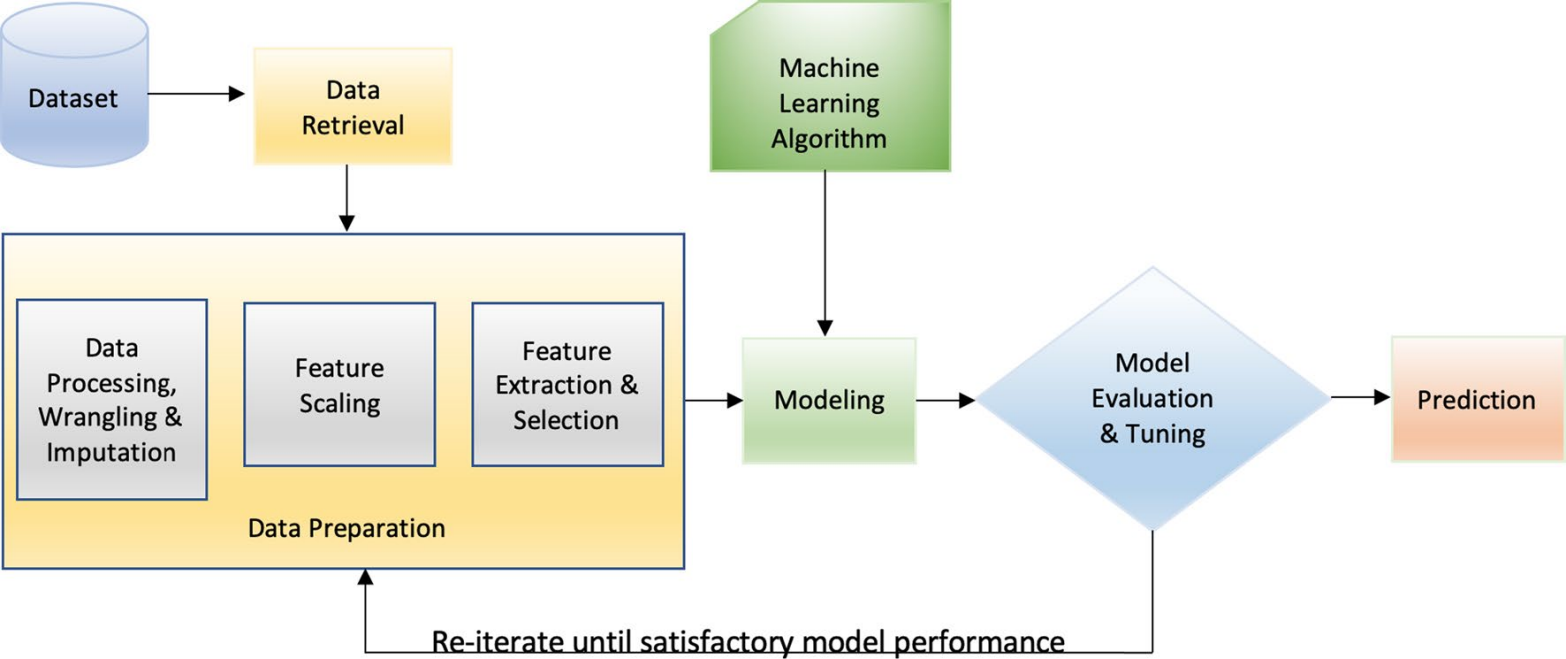
The workflow of the Machine Learning pipeline used in this study

This section describes the ML tasks and the steps undertaken in the experiments. The ML pipeline used in this work is illustrated in Fig. 3. The usage and implementation of all the methods was done in accordance with the journal guidelines and regulations.

### Regression

This task explores the application of different methods to determine which PROMs are optimal for predicting the target outcomes in *dataset 1* and different supervised ML methods to determine the predictability of the outcomes and the best suited algorithm for this task.

Seven algorithms were used to estimate the target outcomes: *Linear Regression* [23], *Passive Aggressive Regression* [24], *Random Forest Regression* [25], *Stochastic Gradient Descent Regression* [26], *AdaBoost Regression* [27], *Support Vector Regression* [28], *XGBoost Regression* [29]. The algorithms were chosen based on the existing literature applying machine learning methods on PROM datasets in a bid to predict patient-specific outcomes [11, 12, 14] and a number of experiments carried out previously where several algorithms were evaluated for their ability to predict patient-reported outcomes, including the ones mentioned above along with Neural Networks, k-NN, Gradient Boosting Machines among others, on similar regression tasks. The evaluation resulted in the selection of the above-mentioned seven algorithms, identified as most suitable for this task.

### Classification

We explored different ML methods to determine which PROMs are most useful for both referral of patients in- and to MBR using *dataset 2*. We used the clinician’s decision as ground truth. Two classifier algorithms: (1) Balanced Random Forest (RF) classifier [30] and (2) Random Under-sampling Boosting classifier (RUSBoost) [31] were chosen because of their ability to deal with class imbalance, handle small data sets and ease of interpretability. Both algorithms create an ensemble of models with a Decision Tree [32] as base estimator, which is also a classifier that has often been used in related work [33–35]. In addition, the respective classifiers were chosen because of their (1) integrated solution to deal with class imbalance; (2) ability to handle mixed data types; (3) ability to perform well with a small sample size (n $\approx$ 1000); (4) high level of model interpretability; and (5) resemblance of thinking compared to a multidisciplinary team of health care professionals [30, 32].

Binary classification tasks were created for the different referral combinations of the 1040 labelled samples, as shown in Table 2. Therefore, each classifier led to six models corresponding to the referral combinations. A nested cross validation was used to evaluate the performance of the models [36]. The nested cross-validation is a nesting of two k-fold cross-validation loops, with k representing the number of folds. The number of folds for both outer and inner loop was chosen to be 5, which is a very common number of folds for cross-validations. In other words, in every loop and for each binary classification task, data was divided into a training dataset with 80% of the samples, and a testing or validation dataset with 20% of the samples.

### Feature selection

Feature selection becomes necessary for datasets with a large number of features to reduce the dimensionality without the loss of any important information. Reducing the dimensionality of the dataset before applying ML methods enables the algorithms to train faster by removing redundant information, thereby reducing the complexity and risk of overfitting the model [37]. Feature selection methods are broadly divided into three types: *filter*, *wrapper*, and *embedded*. *Filter methods* use the principal criteria of the ranking technique for selecting the most relevant features. Features are ranked based on statistical scores, such as correlation, to determine the features’ correlation with the outcome variable. These methods are computationally efficient and do not rely on learning algorithms that can introduce a biased feature subset due to over-fitting [37]. However, a disadvantage of the filter method is that it does not consider the co-linearity among features in the subset. Furthermore, it is difficult to precisely determine the dimension of the optimal feature subset [37]. *Wrapper methods* use the model’s performance metric, for example accuracy, as an objective function to evaluate the feature subset [37]. These methods consider the association among features but are often too computationally expensive to perform an exhaustive search of the feature space. In *Embedded methods*, feature selection is integrated with the training progress of the model to reduce computational time compared to wrapper methods, while still considering the association among features [37, 38]. These methods iteratively extract features that contribute the most to the training for a particular iteration of a model during the training process. Regularisation methods [39, 40] are commonly used embedded methods that penalise a feature based on a coefficient threshold. Feature Importance with ensemble methods is another method to determine impurity-based important features in tree-based algorithms.^2^ Based on the trends observed in the existing literature, it was decided to use mutual information (only in classification task) [41] and impurity-based methods [42, 43] in this work for selecting feature subsets (Additional file 1).

### Hyperparameter optimization

Hyperparameter optimization is useful to find a set of hyperparameters that optimizes the performance of the algorithm [44]. We considered model-based as well as model-free methods for hyperparameter optimization. Model-based optimization methods like Bayesian optimization use a surrogate model and an acquisition function to find the optimal set of hyperparameters [45, 46]. We did not choose model-based optimization since the surrogate model is prone to overfitting on the hyperparameters [47] and this approach is more suitable to models that are computationally expensive to train, such as Deep Neural Networks [46]. Model-free methods can be categorized as heuristic and simple search approaches. Heuristic search approaches maintain a number of hyperparameter sets and use local perturbations and combinations of members in these sets to obtain an improved hyperparameter set [45, 46]. Two common model-free simple search approaches are grid and random search [45]. Grid search is one of the several ways of hyperparameter tuning and entails an exhaustive search through a defined set of hyper-parameter space of a learning algorithm. Random search selects the parameters at random instead of performing an exhaustive search over the hyperparameter space. We used random search in the classification task and grid search in the regression task to tune the hyperparameters of the algorithms  (Additional file 1).

### Evaluation metrics

The evaluation metrics are different for each task owing to the very nature of different approaches undertaken. The evaluation metrics in the regression task are Mean Absolute Error (MAE), R-squared score ($R^2$) and Mean Residual (MR), while for the classification task are Matthews Correlation Coefficient (MCC) [48], Balanced Accuracy (BAC) [49], Sensitivity (SEN) and Specificity (SPE) [50]. MAE is the average of the absolute errors, that is the difference between the observed value and the predicted value. $R^2$ is a goodness-of-fit metric to measure the proportion of variance explained by the independent variable(s) for a dependent variable in a regression model with values in the range [0,1], where 0 implies no observed variance and 1 implies 100% variance in the dependent variable with the movement of the independent variable(s). MR is the average difference between the predicted values and the observed values and is used to determine whether the models are likely to underestimate or overestimate the target value. MCC has a value in the range [− 1, 1] and produces a high score only when the predictions obtain good results in all of the four confusion matrix categories, which is useful for imbalanced classes [51]. The value of BAC lies in the range of [0,1] and is a recommended metric for imbalanced classes [49]. The values of SEN and SPE metrics lie in the range of [0,1] and are used widely to test the performance of binary classification models, where SEN is a measure of the proportion of correctly identified positives (true positive rate) while SPE is a measure of the proportion of correctly identified negatives (true negative rate).

## Experiments and results

The experiments were done in Python [52] using Scikit-learn [53] and Imbalanced-learn [54] (only in classification task). k-fold cross validation is used in the experiments to reduce overfitting and increase the generalizability of the models, with $k=5$ for classification and $k=10$ for regression task.

### Regression task

We used the embedded *feature importance* method of *Random Forest* algorithm to select the relevant features. Four and two features were selected for $PA_{f}$ and $WAI_{f}$, respectively, which were then used to train the ML algorithms mentioned in the “Methods” section. The results are summarised in Table 3.

**Table 3:** Impurity-based feature selection using Random Forest for predicting $PA_{f}$ (a) and $WAI_{f}$ (b)

$PA_{f}$				$WAI_{f}$			
Model	MAE ± SD	${R}^2$	MR	Model	MAE ± SD	${R}^2$	MR
LR	1.54 ± 1.18	0.25	0.050	LR	1.16 ± 1.12	0.27	0.003
PAR	1.54 ± 1.19	0.25	− 0.087	PAR	1.10 ± 1.14	0.28	− 0.288
SGDR	1.55 ± 1.17	0.25	0.143	SGDR	1.10 ± 1.13	0.29	− 0.243
RFR	1.57 ± 1.13	0.25	0.199	**RFR**	**1.09** ± **1.20**	**0.25**	− **0.246**
ABR	1.60 ± 1.14	0.23	0.0	ABR	1.21 ± 1.20	0.18	− 0.090
**SVR**	**1.53** ± **1.15**	**0.27**	**0.102**	SVR	1.11 ± 1.15	0.27	− 0.221
XGB	1.55 ± 1.13	0.26	− 0.015	XGB	1.18 ± 1.12	0.25	0.016

The best performing model are highlighted in bold letters

### Classification task

We used the embedded feature selection method in both classifiers to select optimal features. For each classifier, six binary classification models were trained on different referral combinations, as shown in Table 2. The results are presented in Tables 4 and 5.

**Table 4:** Results for the Balanced Random Forest (RF) classifier (± standard deviation)

	Train	Test			
	MCC	MCC	BAC	SEN	SPE
Model 1: C-P	0.22 ± 0.02	0.14 ± 0.08	0.56 ± 0.04	0.66 ± 0.13	0.47 ± 0.17
Model 2: C-R	0.26 ± 0.01	0.20 ± 0.08	0.60 ± 0.04	0.73 ± 0.11	0.47 ± 0.05
Model 3: P-R	0.22 ± 0.03	0.19 ± 0.06	0.60 ± 0.03	0.59 ± 0.06	0.61 ± 0.02
Model 4: M-C	0.54 ± 0.01	0.46 ± 0.05	0.73 ± 0.03	0.86 ± 0.13	0.59 ± 0.11
Model 5: M-P	0.42 ± 0.02	0.42 ± 0.05	0.70 ± 0.03	0.99 ± 0.03	0.42 ± 0.07
Model 6: M-R	0.53 ± 0.01	0.49 ± 0.06	0.77 ± 0.03	0.98 ± 0.04	0.57 ± 0.05

**Table 5:** Results for the Random Under Sampling Boosting (RUSBoost) classifier ± standard deviation

	Train	Test			
	MCC	MCC	BAC	SEN	SPE
Model 1: C-P	0.22 ± 0.02	0.11 ± 0.07	0.55 ± 0.03	0.72 ± 0.10	0.39 ± 0.13
Model 2: C-R	0.24 ± 0.01	0.21 ± 0.08	0.60 ± 0.04	0.59 ± 0.16	0.61 ± 0.10
Model 3: P-R	0.20 ± 0.02	0.19 ± 0.06	0.60 ± 0.03	0.59 ± 0.06	0.61 ± 0.02
Model 4: M-C	0.55 ± 0.02	0.49 ± 0.10	0.74 ± 0.05	0.94 ± 0.13	0.54 ± 0.10
Model 5: M-P	0.43 ± 0.01	0.43 ± 0.05	0.71 ± 0.03	1.00 ± 0.00	0.42 ± 0.07
Model 6: M-R	0.52 ± 0.01	0.50 ± 0.05	0.78 ± 0.03	0.98 ± 0.03	0.57 ± 0.06

The following observations were made based on the results:

- The overfit is low based on the MCC scores (both classifiers), except for the case Clinic RCR—Polyclinic RCR.
- The cases Polyclinic RMCR—Clinic RCR, Polyclinic RMCR—Polyclinic RCR and Polyclinic RMCR—Rejected show sub-optimal performances with their MCC’s ranging between [0.42, 0.49] for RF and [0.43–0.50] for RUSBoost. Furthermore, their BAC scores are ranging between [0.70, 0.77] for RF and [0.71, 0.78] for RUSBoost.
- The cases Clinic—Rejected, Clinic RCR—Polyclinic RCR and Polyclinic RCR—Rejected all show very poor performances with their MCC’s ranging between [0.14, 0.20] for RF and [0.11, 0.21] for RUSBoost. Furthermore, their BAC scores are ranging between [0.56, 0.60] for RF and [0.55, 0.60] for RUSBoost.

## Discussion

Our experiments on *dataset 1* indicate that ML methods and data science techniques can be used to identify relevant PROMs features and enhance the prediction of patient outcomes, such as pain and work-ability. While in experiments using *dataset 2*, we found that the classifiers perform poorly in predicting treatment referral. These contrasting findings may be attributed to the different predictors available in the two datasets, their strength of association with the target outcomes or the fact that *dataset 1* had the target outcomes measured at baseline while *dataset 2* does not since it’s a one time outcome given by the clinician.

### Clinical relevance

To support shared clinical decision making, it is necessary to build prognostic models that can provide information to clinicians and patients of likely outcomes related to a certain treatment or symptoms profile.

In *dataset 1*, the baseline measurements of the associated target outcomes were their first most important predictors. The superior predictive value of baseline measurements of target outcomes has also been confirmed in other similar studies, such as by Fontana et al. [6] and Huber et al. [14]. In *dataset 2*, the PROMs had low predictive power with regards to referral advises, which is similar to findings in our previous work [5, 55]. Our results again emphasize the difficulty of referring NLBP patients based on PROMs and the need for more research on PROMs to include them in decision support on treatment referral.

### Data science relevance

From a data science perspective, PROM-based analytics is relatively uncharted territory, posing a unique challenge and presenting an opportunity for more research to test the existing methods and develop new ones that can facilitate furthering our comprehension of subjective datasets and their utility in improving patient-centred care. Building a comprehensive view of the patients using data-driven methods and evidence-based research can help clinicians and patients alike get practical insights from the available data to make shared strategic decisions. There is a need to increase awareness, availability, and understanding of subjective patient-centred data to build more sustainable and secure data ecosystems and facilitate a shift towards targeted interventions with the development of diagnostic and prognostic learning models.

## Conclusion and future work

The results presented in this work support our premise that the analytical abilities of ML methods can be leveraged for making predictions using PROMs, given that the PROMs hold predictive power. With better predictors, further development, and thorough validation, ML models can facilitate a shared decision-making process for patients with musculoskeletal disorders in clinical settings. Support Vector Machines, Random Forest, and Random Under-sampling Boosting methods delivered the best performance in the experiments and present promising potential for adaptability and utility in clinical practice. The biggest strength of ML methods is their ability to handle big data and their adaptability to different clinical setups where a certain level of accuracy is required to predict outcomes. There is, however, a need for the development of a standard ML pipeline to guide further research on developing as well as reporting results of ML models that can predict PROMs in other clinical or healthcare datasets with patient-reported outcomes.

## Supplementary Information

**Additional file 1**. Hyperparameters used in the regression models and feature importance of the selected features in the regression and classification models.

## Data Availability

The selfBACK dataset used in the current study is available from the corresponding author on reasonable request. The second dataset analysed in the current study is not publicly available as this data is property of the Roessingh Center of Rehabilitation and the authors do not have permission to share the data further. Further inquiries regarding the second can be directed to Wendy Oude Nijeweme-d’Hollosy.

## Abbreviations

PROMs: Patient-reported outcome measurements
ML: Machine Learning
LBP: Low back pain
NLBP: Neck and/or low back pain
MBR: Multidisciplinary biopsychosocial rehabilitation
DSS: Decision support system
MCID: Minimal clinically important difference
RCT: Randomized controlled trial
FU: Follow-Up
PA: Pain Average
WAI: Workability index
RCR: Roessingh Center of Rehabilitation
HADS: Hospital Anxiety and Depression Scale
MPI: Multidimensional Pain Inventory
PDI: Pain Disability Index
PIPS: Psychological Inflexibility in Pain Scale
RAND: Rand-36 Health Survey
RMCR: Roessingh Medinello Center of Rehabilitation
MAE: Mean Absolute Error
SD: Standard Deviation
MR: Mean Residual
MCC: Mathews Correlation Coefficient
BAC: Balanced Accuracy
SEN: Sensitivity
SPE: Specificity
LR: Linear Regression
PAR: Passive Aggressive Regression
SGDR: Stochastic Gradient Descent Regression
RFR: Random Forest Regression
ABR: AdaBoost Regression
SVR: Support Vector Regression
XGB: XGBoost Regression

## Footnotes

1 https://clinicaltrials.gov/ct2/show/NCT03798288.

2 https://scikit-learn.org/stable/auto_examples/ensemble/plot_forest_importances.html.
