# Supplementary material for: Exploratory application of machine learning methods on patient reported data in the development of supervised models for predicting outcomes
Source: BMC Med Inform Decis Mak. 2022 Sep 1;22:227. doi: 10.1186/s12911-022-01973-9 (PMC9434943; doi:10.1186/s12911-022-01973-9)
Supplement: Supplementary file 1 — Additional file 1. Hyperparameters used in the regression models and feature importance of the selected features in the regression and classification models. [file 12911_2022_1973_MOESM1_ESM.pdf]

Supplementary Material

S1. Hyperparameters

Supplementary Table S1. Hyperparameters used for the development of prediction models in the regression task.

Model	PA_f	WAI_f
Linear Regressor	copy_X=True, fit_intercept= False, normalize=True	copy_X=True, fit_intercept= True, normalize=True
Passive Aggressive Regressor	C= 0.01, early_stopping=True, epsilon= 0.01, n_iter_no_change=500, loss= 'squared_epsilon_insensitive', validation_fraction=0.2	C= 0.1, early_stopping=True, epsilon= 0.001, n_iter_no_change=500, loss= 'squared_epsilon_insensitive', validation_fraction=0.1
Stochastic Gradient Descent Regressor	loss='epsilon_insensitive', penalty='l2', alpha=0.001, l1_ratio=0.15, fit_intercept=True, max_iter=1000, tol=0.001, shuffle=False, verbose=0, epsilon=0.01, random_state=42, learning_rate='adaptive', eta0= 1, power_t=0.25	loss='epsilon_insensitive', penalty='l1', alpha=1e-07, l1_ratio=0.15, fit_intercept=True, max_iter=1000, tol=1e-07, shuffle=True, verbose=0, epsilon=0.1, random_state=42, learning_rate='invscaling', eta0=0.1, power_t=0.25
Random Forest Regressor	n_estimators=50, max_depth=2, criterion='mae', oob_score=False,	n_estimators=50, max_depth=2, criterion='mae', oob_score=False
AdaBoost Regressor	n_estimators=100, learning_rate=0.01, loss='exponential'	n_estimators=10, learning_rate=0.001, loss='exponential'
Support Vector Regressor	kernel='poly', degree=2, tol=0.0001 , C=1, epsilon=0.1, verbose=False, max_iter=1000	kernel='poly', degree=2, tol=1e-05 , C=1, epsilon=0.001, verbose=False, max_iter=1000
XGBoost Regressor	learning_rate=0.1, max_depth=2, n_estimators=100, objective='reg:squarederror', reg_alpha=0.015	learning_rate=0.5, max_depth=2, n_estimators=10, objective='reg:squarederror', reg_alpha=0.9

S2. Feature Importance

S2.1. Regression Models

Supplementary Table S2.1.1. Feature importance of the selected features of PA_f in the prediction models.

Feature Importances: PA_f				
**Model \ Feature**	**BIPQ symptoms**	**Baseline PA**	**EQ5D**	**Sleep end**
Linear Regressor	0.41	0.26	-0.09	0.06
Passsive Aggressive Regressor	0.29	0.18	-0.21	0.04
Stochastic Gradient Descent Regressor	0.30	0.10	-0.17	0.08
Random Forest Regressor	0.33	0.28	0.17	0.21
AdaBoost Regressor	0.42	0.18	0.27	0.11
XGBoost Regressor	0.37	0.17	0.14	0.29

Supplementary Table S2.1.2. Feature importance of the selected features of WAI_f in the prediction models.

Feature Importances: WAI_f		
**Model \ Feature**	**Baseline WAI**	**Pain self efficacy**
Linear Regressor	0.39	0.18
Passsive Aggressive Regressor	0.47	0.14
Stochastic Gradient Descent Regressor	0.32	0.21
Random Forest Regressor	0.80	0.19
AdaBoost Regressor	0.71	0.28
XGBoost Regressor	0.89	0.10

S2.2. Classification Models

Supplementary Table S2.2.1. Feature importance of the selected features in the classification models.

Feature Importances							
**Questionnaire**	**Features**	**Medinello - Clinic**	**Medinello - Polyclinic**	**Medinello - Rejected**	**Clinic - Rejected**	**Clinic - Polyclinic**	**Polyclinic - Rejected**
HADS	Anxiety	0.04	0.09	0.06	0.04	0.02	0.05
HADS	Depression	0.14	0.14	0.16	0.05	0.04	0.07
HADS	Total score	0.42	0.54	0.56	0.05	0.04	0.08
PDI	Total score	0.04	0.01	0.01	0.04	0.10	0.05
MPI	Pain severity	0.01	0.01	0.00	0.02	0.02	0.02
MPI	Interference	0.04	0.01	0.01	0.03	0.10	0.04
MPI	Life control	0.02	0.03	0.02	0.03	0.02	0.05
MPI	Affective distress	0.01	0.01	0.01	0.03	0.02	0.04
MPI	Support	0.01	0.01	0.01	0.04	0.01	0.04
MPI	Punishing responses	0.01	0.00	0.00	0.03	0.02	0.03
MPI	Solicitous responses	0.01	0.00	0.00	0.04	0.03	0.04
MPI	Distractive responses	0.01	0.00	0.01	0.05	0.02	0.04
MPI	Household chores	0.01	0.01	0.00	0.07	0.09	0.03
MPI	Outdoor work	0.02	0.01	0.00	0.02	0.02	0.02
MPI	Social activities	0.02	0.01	0.03	0.06	0.02	0.07
MPI	General activities	0.01	0.01	0.02	0.10	0.05	0.06
PIPS	Avoidance	0.01	0.03	0.02	0.04	0.02	0.05
PIPS	Cognitive fusion	0.02	0.01	0.00	0.03	0.03	0.03
PIPS	Total score	0.02	0.02	0.01	0.03	0.02	0.04
RAND36	Physical functioning	0.02	0.01	0.01	0.04	0.11	0.05
RAND36	Role limitations	0.00	0.00	0.00	0.01	0.01	0.00
RAND36	Mental health	0.04	0.03	0.06	0.03	0.04	0.07
RAND36	Vitality	0.05	0.01	0.00	0.11	0.17	0.03
